# Residual Inflammatory and Cholesterol Risk and the Association With Recurrent Cardiovascular Events in East Asian Patients After Percutaneous Coronary Intervention

**DOI:** 10.31083/RCM36438

**Published:** 2025-09-19

**Authors:** Ang Gao, Tingting Guo, Zhiqiang Yang, Hong Qiu, Runlin Gao

**Affiliations:** ^1^Department of Cardio-Metabolic Medicine Center, Fuwai Hospital, National Center for Cardiovascular Diseases, Chinese Academy of Medical Sciences and Peking Union Medical College, 100006 Beijing, China; ^2^Intensive Care Center, Fuwai Hospital, National Center for Cardiovascular Diseases, Chinese Academy of Medical Sciences and Peking Union Medical College, 100006 Beijing, China; ^3^Department of Cardiology, Tibet Autonomous Region People’s Hospital, 850002 Lasa, Tibet Autonomous Region, China; ^4^Department of Cardiology, Fuwai Yunnan Hospital, Chinese Academy of Medical Sciences, Affiliated Cardiovascular Hospital of Kunming Medical University, 650000 Kunming, Yunnan, China; ^5^Department of Cardiology, Coronary Artery Disease Center, Fuwai Hospital, National Center for Cardiovascular Diseases, Chinese Academy of Medical Sciences and Peking Union Medical College, 100006 Beijing, China

**Keywords:** percutaneous coronary intervention, East Asian, residual inflammatory risk, residual cholesterol risk, major adverse cardiovascular and cerebrovascular event

## Abstract

**Background:**

The applicability of currently established high-risk inflammatory criteria to East Asian patients is unknown, particularly concerning the hypersensitive C-reactive protein (hs-CRP) cutoff value. In addition, the role of cholesterol and inflammation in determining the prognosis of these patients might shift after the patient accepts lipid-lowering treatments. This study aimed to explore the high-risk hs-CRP cutoff value and compare the prognostic value between inflammation and cholesterol risk in the East Asian population after treatment with percutaneous coronary intervention (PCI).

**Methods:**

Post-PCI patients with serial hs-CRP and low-density lipoprotein cholesterol (LDL-C) level measurements were retrospectively enrolled. Major adverse cardiovascular and cerebrovascular events (MACCEs) were defined as a composite of cardiovascular death, non-fatal acute myocardial infarction (AMI), non-fatal stroke, and unplanned coronary revascularization. The association between residual risks and MACCEs was evaluated.

**Results:**

During a median follow-up of 30.4 months, 403 MACCEs occurred among 2373 patients. The high-risk LDL-C and hs-CRP cutoff values in the present study were set at 1.56 mg/L and 1.80 mmol/L, respectively, based on the results of tertile stratification and restricted cubic spline analysis. The adjusted hazard ratios (HRs) and 95% confidence intervals (CIs) of residual cholesterol risk (hs-CRP <1.56 mg/L; LDL-C ≥1.80 mmol/L), residual inflammatory risk (hs-CRP ≥1.56 mg/L; LDL-C <1.80 mmol/L), and residual cholesterol and inflammatory risk (hs-CRP ≥1.56 mg/L; LDL-C ≥1.80 mmol/L) for MACCEs were 1.26 (0.95–1.66), 2.15 (1.57–2.93), and 2.07 (1.55–2.76), respectively. Inflammatory-induced MACCEs were more likely to be associated with the increased risk of non-fatal AMI (HR: 4.48; 95% CI: 2.07–9.73; *p *< 0.001), while cholesterol-induced MACCEs were more likely to be associated with the increased risk of non-target vessel revascularization (TVR: HR: 1.60; 95% CI: 1.08–2.37; *p* = 0.019). Persistent high inflammatory risk (baseline and follow-up hs-CRP ≥1.56 mg/L) can be a major determinant of MACCEs (adjusted HR: 2.03; 95% CI: 1.64–2.52; *p *< 0.001), while persistent high cholesterol risk (baseline and follow-up LDL-C ≥1.80 mmol/L) was not. Serial hs-CRP measurements could produce more predictive values for MACCEs than a single measurement.

**Conclusions:**

Despite statin treatment, residual cholesterol and inflammatory risks persist in post-PCI patients. The high-risk hs-CRP standard may be lower in East Asian patients than their Western counterparts, with a cutoff value of 1.56 mg/L. Inflammation and cholesterol could be major determinants for recurrent cardiovascular events, while hs-CRP seems to be a stronger predictor than LDL-C in post-PCI patients receiving statin therapy.

**Clinical Trial Registration:**

ChiCTR2100047090, https://www.chictr.org.cn/showproj.html?proj=127821.

## 1. Introduction

Due to the aging population and the increasing prevalence of cardiometabolic risk factors, cardiovascular deaths have become the leading cause of mortality in China [1]. Statin has been recognized as the cornerstone of secondary prevention due to its effectiveness in lowering the rates of recurrent myocardial infarction, stroke and cardiovascular death [2]. However, statin-treated patients, especially those with advanced atherosclerotic cardiovascular disease (ASCVD), still suffer from a relatively high incidence of recurrent events even after an early revascularization strategy, an issue commonly ascribed to the problem of ‘residual risk’ [3]. Residual cholesterol risk (RCR) and residual inflammatory risk (RIR) were both shown to be important predictors for the prognosis of ASCVD patients and therapies targeting cholesterol and inflammation delivered positive results [4, 5, 6, 7, 8]. However, there is a lack of serial monitoring of hypersensitive C-reactive protein (hs-CRP) and low-density lipoprotein cholesterol (LDL-C) values during medical follow-up. Therefore, the relative importance of inflammatory and cholesterol risk for predicting recurrent adverse clinical events after accepting statins remains elusive. In addition, the inflammatory burden, especially in East Asian patients, is generally lower than their counterparts in Western populations [9, 10, 11]. Whether the established standard (hs-CRP ≥2 mg/L) used for evaluating RIR could also be applied in East Asian patients is still unknown. Limited studies concentrated on the increased risk for recurrent cardiovascular events caused by inflammation in post-percutaneous coronary intervention (PCI) patients with achieved LDL-C levels. Hence, this study aimed to explore the high-risk hs-CRP cutoff value and compare the prognostic value of inflammation and cholesterol in East Asian population after PCI treatment.

## 2. Methods

### 2.1 Data Collection and Disease Definition

The data about enrolled patients were derived from the efficacy and safety of genetic and platelet function testing for guiding antiplatelet therapy after percutaneous coronary intervention (GF-APT) registry (ChiCTR2100047090). The GF-APT was a single-center registry, which retrospectively enrolled consecutive PCI-treated patients during the hospitalization and discharged with dual antiplatelet therapy in the Fuwai Hospital between January 2016 and December 2018. The GF-APT registry was designed to explore whether the genetic-guided selection of an oral P2Y purinoceptor 12 (P2Y12) inhibitor therapy would be beneficial for patients after PCI treatment. In the GF-APT, demographics data, medical history, results of laboratory tests, angiographic features, procedural characteristics, and information on treatment outcomes were collected from electronic medical records for all enrolled patients. The primary efficacy endpoint of the study was a composite of cardiac death, myocardial infarction, and unplanned coronary revascularization following the index PCI. The major exclusion criteria of the registry were as follows: (1) expected duration of dual antiplatelet therapy <6 months (2) indications for long-term treatment with oral anticoagulants, (3) life expectancy of <1 year, (4) any contraindication to aspirin or P2Y12 receptor inhibitors, including ticagrelor and clopidogrel. This study has been approved by the institutional ethics committee of Fuwai Hospital (No. 2021-1063) and was performed in accordance with the Principles of the Declaration of Helsinki. All participants signed the written informed consent before discharge. Demographic data and medication at discharge were obtained through a review of the medical records, which was approved by the Fuwai Hospital. Blood samples were taken after overnight fasting if participants were not indicated for emergent coronary revascularization. On-admission biochemical labs were collected via the cubital vein within 24 hours following hospital admission. For patients receiving emergency PCI, additional blood sampling was performed at admission. At a median of 2-month (2 months ± 1 month) visits, follow-up biochemical measurements were obtained from blood samples taken from the cubital vein after overnight fasting. The hs-CRP level was measured via the Beckman Assay 360 clinical chemistry analyzer (Beckman Coulter, Brea, CA, USA). Plasma levels of lipid profile, including total cholesterol (TC), LDL-C, triglyceride (TG) and high-density lipoprotein cholesterol (HDL-C) were measured by an automatic biochemistry analyzer (Kyowa, Tokyo, Japan), with a coefficient of variation of <5% and a total imprecision of <10%. LDL-C was calculated using the Friedewald formula from TC, HDL-C, and TG. All enrolled participants were followed up for at least 12 months or until the time of a major adverse clinical event. Follow-up was performed by telephone interviewers using standardized questionnaires at 6 and 12 months after the PCI treatment and then the follow-up was recorded by the clinical visit using hospital medical record system. The intensity of statin treatments was defined according to ACC/AHA guideline definitions [12]. The diagnosis of diabetes mellitus was based on the previous diagnosis and treatment with glucose-lowering medication or recommendations from the American Diabetes Association [13]. Hypertension was defined by the recommendations from the European Society of Hypertension, an office systolic blood pressure value ≥140 mmHg or a diastolic blood pressure value ≥90 mmHg or the use of antihypertensive drugs in the past 2 weeks [14]. Dyslipidemia was characterized by increased total cholesterol, LDL-C or triglyceride level or a decreased high-density lipoprotein cholesterol level according to the third report of the National Cholesterol Education Program [15]. Acute myocardial infarction was defined as increased cardiac troponin values with ischemic symptoms or ischemic changes on an electrocardiogram, imaging evidence of recent loss of viable myocardium or new regional wall motion abnormalities that were not related to the procedure [16]. The characteristics of coronary artery lesions are defined on the basis of the ACC/AHA guidelines for coronary lesion classification [17]. Multivessel disease was defined as a ≥50% diameter stenosis occurring in 2 or more vessels.

### 2.2 Clinical Outcomes Definition and Adjudication

The primary endpoint of this study was the occurrence of MACCE after PCI treatment, defined as the composite of cardiovascular death, non-fatal acute myocardial infarction (AMI), non-fatal stroke and unplanned coronary revascularization. Non-fatal AMI was adjudicated using the universal definition (Fourth Universal Definition of MI). The definition of non-fatal stroke should include: (1) acute neurological deficit lasting >24 hours; (2) Neuroimaging confirmation by CT/MRI; (3) Absence of death within 30 days [18]. Unplanned coronary revascularization was defined according to the 2018 ESC/EACTS guidelines on myocardial revascularization [19]: (1) Any PCI or coronary artery bridge grafting (CABG) not pre-scheduled during index hospitalization and not part of staged procedures; (2) Triggered by either recurrent angina with objective ischemia or acute coronary syndrome (ACS); (3) Adjudicated by an independent clinical events committee.

### 2.3 Statistical Analysis

**Fig. 1.**
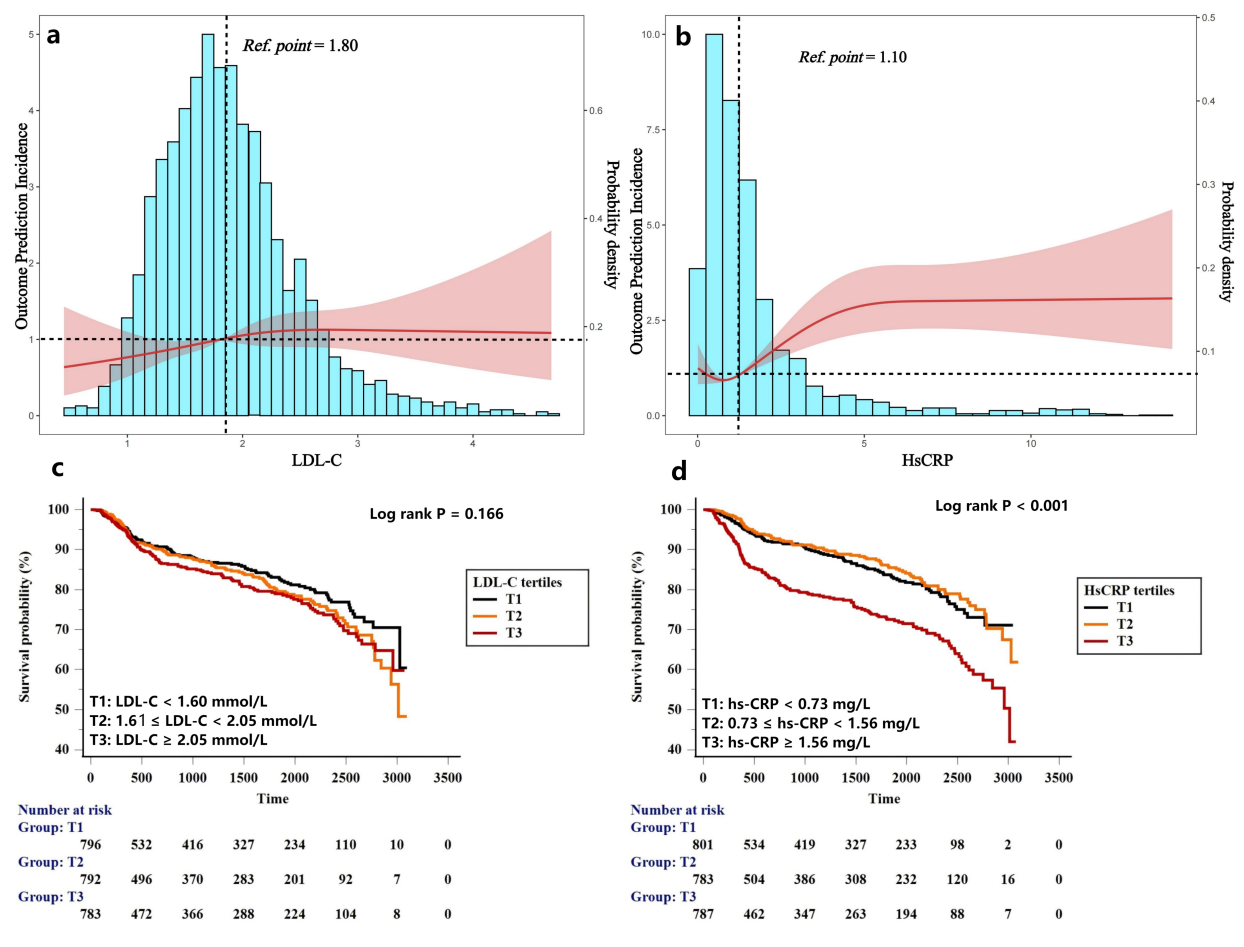
**Distribution of LDL-C and hs-CRP levels among PCI-treated patients and RCS plots for the association with MACCEs (a,b). Kaplan-Meier curves for MACCEs based on tertiles of LDL-C and hs-CRP levels (c,d)**. LDL-C, low-density lipoprotein cholesterol; hs-CRP, hypersensitive C-reactive protein; PCI, percutaneous coronary intervention; RCS, restricted cubic spline; MACCE, Major adverse cardiovascular and cerebrovascular event.

To better understand the characteristics of PCI-treated patients with residual cholesterol and inflammatory burdens, participants were categorized into four groups according to the high-risk LDL-C and hs-CRP values. To explore the high-risk cutoff values of hs-CRP and LDL-C for recurrent cardiovascular events, we performed restricted cubic spline analysis, which showed linear relationships between follow-up LDL-C and hs-CRP levels and risk of MACCE with an LDL-C ≥1.80 mmol/L and hs-CRP ≥1.10 mg/L (Fig. 1a,b). Participants were further divided into 3 tertiles according to the follow-up hs-CRP value (T1: hs-CRP ≤0.73 mg/L; T2: 0.73 < hs-CRP < 1.56 mg/L; T3: hs-CRP ≥1.56 mg/L) and LDL-C value (T1: LDL-C <1.60 mmol/L; T2: 1.61 ≤ LDL-C < 2.05 mmol/L; T3: LDL-C ≥2.05 mmol/L). During the follow-up period, Kaplan-Meier curves (Fig. 1c,d) showed differences in the risk of MACCEs between tertiles of follow-up hs-CRP but not in the LDL-C tertiles. Table 1 shows the predictive value of follow-up hs-CRP and LDL-C tertiles for the risk of MACCE. Compared with the lowest tertile, only the highest tertile of hs-CRP showed significant association with MACCE (hs-CRP: T3 versus T1, adjusted hazard ratio (HR) 1.67, 95% confidence interval (CI) 1.30–2.14, *p* < 0.001; LDL-C: T3 versus T1, adjusted HR 1.17, 95% CI 0.91–1.50, *p* = 0.224). To determine whether hs-CRP 1.10 mg/L or 1.56 mg/L would be the optimal cutoff value, we divided the patients into 4 groups (Group 1: hs-CRP <1.1 mg/L, Group 2: 1.1 ≤ hs-CRP < 1.56 mg/L, Group 3: 1.56 ≤ hs-CRP < 2 mg/L, Group 4: hs-CRP ≥2 mg/L) and conducted a survival analysis. The results are shown in the **Supplementary Fig. 1**. Compared with Group 1, the risk of MACCE was not significantly increased in Group 2 (HR 0.99, 95% CI 0.73–1.35, *p* = 0.962). Differences could be observed in the risk of MACCE between Group 3 (HR 1.42, 95% CI 1.01–2.01, *p* = 0.045) and Group 4 (HR 2.23, 95% CI 1.79–2.80, *p* < 0.001) if Group 1 was designated as the reference group for comparison. **Supplementary Fig. 2** indicated that an increased risk of MACCE in patients with LDL-C ≥1.80 mmol/L. Therefore, the high-risk LDL-C and hs-CRP cutoff value in the present study were set as 1.80 mmol/L and 1.56 mg/L. Descriptive variables were expressed as the mean ± standard deviation or median with interquartile range. Categorical variables were presented as frequencies and percentages, and the differences between groups were determined by one-way analysis of variance or the Kruskal-Wallis H test for normally or nonnormally distributed variables. Cumulative event rates were compared using the log-rank test, and the Kaplan-Meier method was used to depict the time-to-event curves. The associations between cholesterol or inflammatory risk and MACCE were determined using a multivariable Cox regression model after adjustment. A two-sided *p* value < 0.05 was considered statistically significant. Statistical analyses were conducted using R version 4.4.1 (R Foundation for Statistical Computing, Vienna, Austria) and SPSS 26.0 (IBM Corp., Armonk, NY, USA).

**Table 1.** **Predictive value of follow-up hs-CRP and LDL-C tertiles for MACCE risks**.

Follow-up		MACCE events	Unadjusted model			Adjusted model		
		n/N	HR	95% CI	*p* value	HR	95% CI	*p* value
Hs-CRP tertiles								
	T1 (T1 <0.73)	111/801	*Ref.*	*Ref.*		*Ref.*	*Ref.*	
	T2 (0.73 ≤ T2 < 1.56)	100/783	0.89	0.68–1.16	0.380	0.80	0.61–1.05	0.111
	T3 (T3 ≥1.56)	192/789	1.91	1.51–2.42	< **0.001**	1.67	1.30–2.14	< **0.001**
LDL-C tertiles								
	T1 (T1 <1.60)	124/797	*Ref.*	*Ref.*		*Ref.*	*Ref.*	
	T2 (1.61 ≤ T2 < 2.05)	135/793	1.17	0.92–1.50	0.203	1.13	0.88–1.46	0.329
	T3 (T3 ≥2.05)	144/783	1.26	0.99–1.60	0.062	1.17	0.91–1.50	0.224

Adjusted model included age, male sex, body mass index, current smoker, index presentation for PCI, medical history of previous myocardial infarction, coronary revascularization, hypertension, Type 2 diabetes mellitus, left ventricular ejection fraction, multivessel disease, ACC/AHA defined type B2/C lesions, stent length, use of ticagrelor and angiotensin blockade at discharge. *p* value in bold indicate the differences between groups were statistically significant. Ref, reference.

## 3. Results

### 3.1 Study Design and Populations

**Fig. 2.**
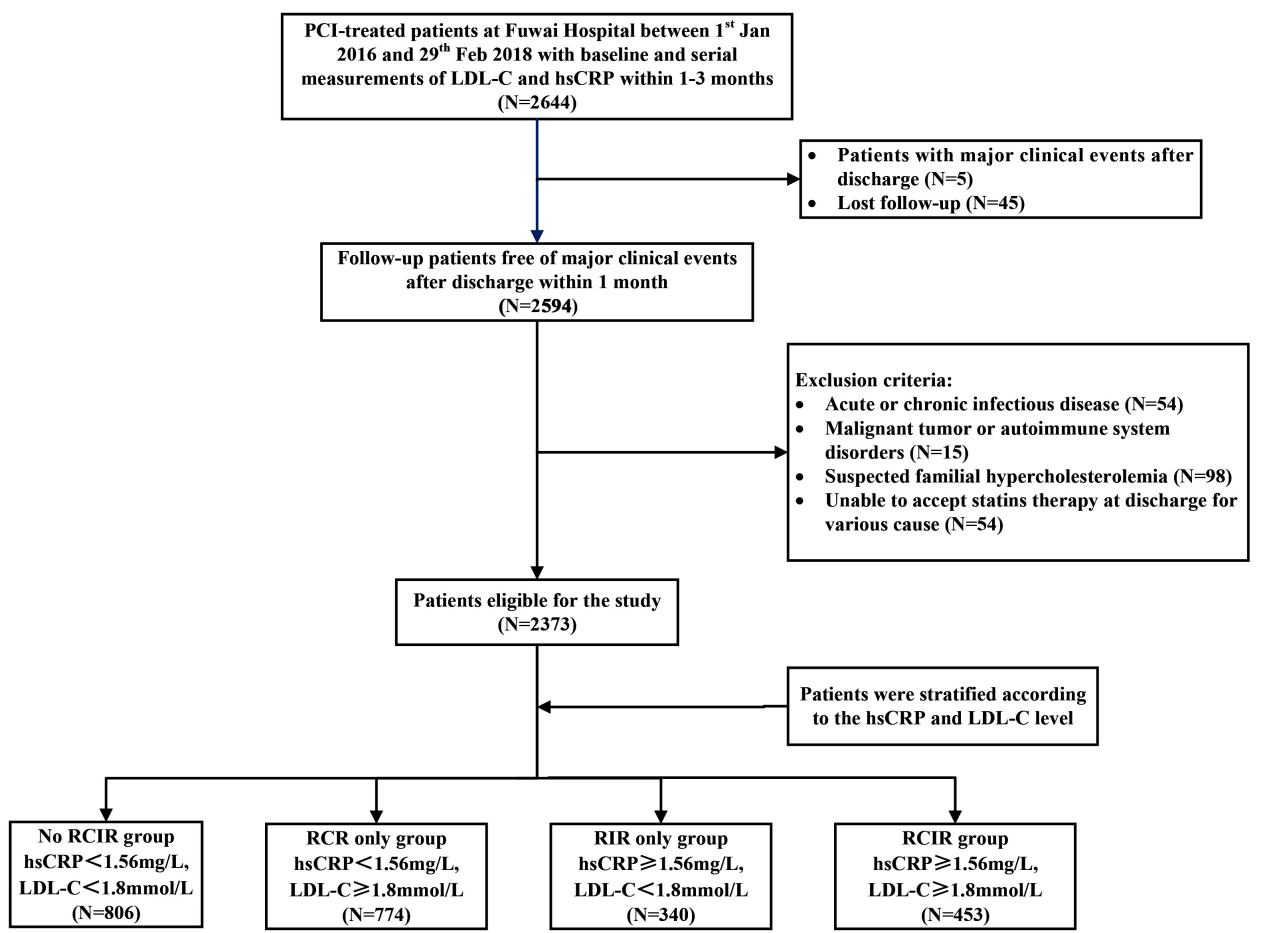
**Flow diagram of patient selection**. RCIR, residual cholesterol and inflammatory risk; RCR, residual cholesterol risk; RIR, residual inflammatory risk.

A total of 2644 consecutive participants with serial monitoring of hs-CRP and LDL-C values were recruited into study. The exclusion criteria included: (1) Failure to complete at least a 12-month follow-up (N = 45); (2) Major adverse clinical events before the latest measurement within 3 months after PCI procedure (N = 5); (3) Acute or chronic infectious diseases (N = 54); (4) Malignant tumors or autoimmune system disorders (N = 15); (5) Suspected familial hypercholesterolemia (N = 98); (6) Unable to accept statin therapy at discharge (N = 54). Finally, 2373 participants were included in the analysis. The patients were classified into 4 groups according to high-risk LDL-C and hs-CRP cutoff values: no residual cholesterol and inflammatory risk (RCIR) group: hs-CRP <1.56 mg/L, LDL-C <1.80 mmol/L (N = 806); RCR only group: hs-CRP <1.56 mg/L, LDL-C ≥1.80 mmol/L (N = 774); RIR only group: hs-CRP ≥1.56 mg/L, LDL-C <1.80 mmol/L (N = 340); RCIR group: hs-CRP ≥1.56 mg/L, LDL-C ≥1.80 mmol/L (N = 453). Fig. 2 showed the detailed flow chart of the study. Clinical characteristics of enrolled patients were shown in Table 2. The mean age of the enrolled patients was 58.5 ± 10.32 years. More than a half of patients (68.4%) presented with ACS on admission and most were male (76.4%). Hs-CRP and LDL-C values were all significantly decreased from 1.7 (IQR: 0.9–4.2) mg/L and 2.4 ± 0.82 mmol/L at admission to 1.1 (IQR: 0.6–1.97) mg/L and 1.9 ± 0.60 mmol/L at follow-up (all *p* < 0.001). During a median of 30.4 months follow-up, 403 of the enrolled cohort (17.0%) experienced MACCE (4 cardiovascular deaths [0.2%], 73 non-fatal AMI [3.1%], 358 unplanned coronary revascularization [15.1%] and 10 non-fatal strokes [0.4%]).

**Table 2.** **Baseline characteristics of enrolled patients stratified by residual inflammatory and cholesterol risk**.

		Overall	no RCIR	RCR only	RIR only	RCIR	*p* value
		(n = 2373)	(n = 806)	(n = 774)	(n = 340)	(n = 453)	
Demographic data							
	Age (years)	58.5 ± 10.32	57.2 ± 10.36	59.4 ± 10.04	59.3 ± 10.78	58.7 ± 10.18	< **0.001**
	Male sex, n (%)	1813 (76.4%)	650 (80.8%)	586 (75.7%)	255 (75.0%)	322 (71.1%)	**0.001**
Cardiovascular risk factors							
	Hypertension, n (%)	1517 (63.9%)	475 (58.9%)	484 (62.5%)	241 (70.9%)	317 (70.0%)	< **0.001**
	Diabetes mellitus, n (%)	855 (36.0%)	262 (32.5%)	268 (34.6%)	132 (38.8%)	193 (42.6%)	**0.002**
	Hyperlipidemia, n (%)	2165 (91.2%)	727 (90.2%)	707 (91.3%)	305 (89.7%)	426 (94.0%)	0.088
	Current Smokers, n (%)	1418 (59.8%)	483 (60.0%)	445 (57.5%)	216 (63.5%)	274 (60.5%)	0.287
	Body mass index (Kg/m^2^)	25.8 ± 3.41	25.6 ± 3.11	25.4 ± 3.32	26.2 ± 3.57	26.7 ± 3.74	< **0.001**
Previous medical history							
	Previous coronary revascularization, n (%)	445 (18.8%)	138 (17.1%)	161 (20.8%)	53 (15.6%)	93 (20.5%)	0.080
	Previous myocardial infarction, n (%)	308 (13.0%)	105 (13.0%)	108 (13.9%)	33 (9.7%)	62 (13.7%)	0.253
	Previous stroke, n (%)	249 (10.5%)	71 (8.8%)	92 (11.9%)	38 (11.1%)	48 (10.6%)	0.240
Index presentation							**0.007**
	Stable angina	749 (31.6%)	257 (31.9%)	263 (34.0%)	96 (28.2%)	133 (29.4%)	
	NSTE-ACS	1188 (50.1%)	389 (48.3%)	395 (51.0%)	162 (47.6%)	242 (53.4%)	
	STEMI	436 (18.3%)	160 (19.9%)	116 (15.0%)	82 (24.1%)	78 (17.2%)	
Laboratory measurements							
	White blood cell count, 10^9^/L	7.1 ± 2.12	6.5 ± 1.46	6.1 ± 1.96	7.3 ± 1.71	7.2 ± 1.86	< **0.001**
	Hemoglobin, g/dL	14.3 ± 1.62	14.3 ± 1.48	13.0 ± 1.35	13.9 ± 1.67	13.8 ± 1.62	< **0.001**
	Baseline hs-CRP, mg/L	1.7 (0.9–4.2)	1.2 (0.7–2.8)	1.3 (0.7–2.8)	3.2 (1.7–7.8)	3.1 (1.8–7.6)	< **0.001**
	Follow-up hs-CRP, mg/L	1.1 (0.6–1.97)	0.7 (0.4–1.0)	0.8 (0.4–1.2)	2.6 (1.9–4.6)	2.8 (2.0–4.6)	< **0.001**
	Creatinine, mmol/L	80.8 ± 16.13	81.9 ± 15.32	81.9 ± 17.29	85.0 ± 20.95	80.4 ± 16.65	**0.002**
	Fasting blood glucose, mmol/L	6.3 ± 2.22	5.9 ± 1.61	6.1 ± 1.58	6.2 ± 1.74	6.5 ± 2.24	< **0.001**
	HbA1c, %	6.3 ± 1.22	6.2 ± 1.17	6.2 ± 1.15	6.4 ± 1.32	6.5 ± 1.30	< **0.001**
	Triglyceride, mmol/L	1.7 ± 0.98	1.3 ± 0.62	1.5 ± 0.70	1.5 ± 0.87	1.7 ± 0.80	< **0.001**
	Total cholesterol, mmol/L	4.1 ± 0.98	2.9 ± 0.41	3.9 ± 0.59	2.9 ± 0.43	4.0 ± 0.70	< **0.001**
	HDL-C, mmol/L	1.1 ± 0.37	1.1 ± 0.27	1.2 ± 0.28	1.0 ± 0.28	1.1 ± 0.27	< **0.001**
	Baseline LDL-C, mmol/L	2.4 ± 0.82	2.1 ± 0.74	2.6 ± 0.78	2.2 ± 0.78	2.8 ± 0.82	< **0.001**
	Follow-up LDL-C, mmol/L	1.9 ± 0.60	1.4 ± 0.26	2.3 ± 0.45	1.4 ± 0.25	2.4 ± 0.55	< **0.001**
	LVEF, %	61.2 ± 7.55	61.5 ± 6.97	61.3 ± 7.56	60.2 ± 7.46	60.9 ± 7.54	**0.040**
Medication at discharge							
	Aspirin + Ticagrelor, n (%)	521 (22.0%)	199 (24.7%)	123 (18.9%)	85 (25.0%)	114 (25.2%)	< **0.001**
	β-blockers, n (%)	2052 (86.5%)	698 (86.6%)	664 (85.8%)	291 (85.6%)	399 (88.1%)	0.671
	ACEI/ARB, n (%)	1347 (56.8%)	447 (55.5%)	430 (55.6%)	204 (60.0%)	266 (58.7%)	0.365
Statins intensity							0.079
	Low or middle-intensity, n (%)	1886 (79.5%)	627 (78.7%)	632 (80.7%)	259 (79.1%)	368 (79.1%)	
	High-intensity or plus Ezetimibe, n (%)	487 (20.5%)	179 (21.3%)	142 (19.3%)	81 (20.9%)	85 (20.9%)	
Target vessel during PCI, n (%)							
	LMCA, n (%)	131 (5.5%)	42 (5.2%)	41 (5.3%)	21 (6.2%)	27 (6.0%)	0.880
	LAD, n (%)	1392 (58.7%)	484 (60.0%)	450 (58.1%)	205 (60.3%)	253 (55.8%)	0.461
	LCX, n (%)	599 (25.2%)	184 (22.8%)	213 (27.5%)	76 (22.3%)	126 (27.8%)	0.052
	RCA, n (%)	840 (36.4%)	291 (36.1%)	259 (33.5%)	124 (36.5%)	166 (36.6%)	0.589
	Others, n (%)	8 (0.3%)	0 (0.0%)	5 (0.6%)	2 (0.6%)	1 (0.2%)	0.062
Multivessel disease, n (%)		1703 (71.8%)	550 (68.2%)	559 (72.2%)	253 (74.4%)	341 (75.3%)	**0.030**
Stent length, mm		36.3 ± 25.00	36.4 ± 23.84	36.4 ± 25.89	36.9 ± 25.93	35.6 ± 26.21	0.900
AHA/ACC lesion: type B2/C, n (%)		1726 (72.7%)	589 (73.1%)	567 (73.3%)	242 (71.2%)	328 (72.4%)	0.898
Major adverse clinical events, n (%)		403 (17.0%)	94 (11.7%)	117 (15.1%)	83 (24.4%)	110 (24.3%)	< **0.001**
	Cardiovascular death, n (%)	4 (0.2%)	0 (0.0%)	0 (0.0%)	3 (0.9%)	1 (0.2%)	**0.004**
	Non-fatal AMI, n (%)	73 (3.1%)	11 (1.4%)	17 (2.2%)	19 (5.6%)	26 (5.7%)	< **0.001**
	Non-fatal Stroke, n (%)	10 (4.3%)	1 (0.1%)	2 (0.3%)	1 (0.3%)	6 (1.3%)	**0.021**
	Target vessel revascularization, n (%)	152 (6.4%)	44 (5.5%)	39 (5.0%)	26 (7.6%)	43 (9.5%)	< **0.001**
	Non-target vessel revascularization, n (%)	206 (8.7%)	44 (5.5%)	66 (8.5%)	46 (13.5%)	50 (11.0%)	< **0.001**

Data are expressed as the mean ± SD, median with interquartile range or n (%). *p* values in bold indicate the differences between groups were statistically significant. Abbreviations: NSTE-ACS, non-ST-segment elevation acute coronary syndrome; STEMI, ST-segment elevation myocardial infarction; HbA1c, hemoglobin A1c; HDL-C, high-density lipoprotein cholesterol; LVEF, left ventricular ejection fraction; ACEI/ARB, angiotensin converting enzyme inhibitor/angiotensin receptor blocker; LMCA, left main coronary artery; LAD, left anterior descending coronary artery; LCX, left circumflex coronary artery; RCA, right coronary artery; AMI, acute myocardial infarction.

### 3.2 Distribution of Enrolled Patients Regarding Residual Cholesterol and Inflammatory Risk

According to the high-risk hs-CRP and LDL-C cutoff value, the cohort were classified into no RCIR group _(hs-CRP <1.56 mg/L and LDL-C <1.80 mmol/L, N = 806, 38.7%)_, RCR only group _(hs-CRP <1.56 mg/L and LDL-C ≥1.80 mmol/L, N = 774, 37.2%)_, RIR only group _(hs-CRP ≥1.56 mg/L and LDL-C <1.80 mmol/L, N = 340, 9.9%)_ and RCIR group _(hs-CRP ≥1.56 mg/L and LDL-C ≥1.80 mmol/L, N = 453, 14.2%)_. Table 2 showed that compared with those with no RCIR, patients with residual risks had more rates of hypertension, diabetes mellitus, and higher body mass index (BMI) values. The prevalence of MACCE was higher in RCR only (15.1%), RIR only (24.1%) and RCIR (24.3%) group than in the no RCIR group (*p* < 0.001). **Supplementary Fig. 3** shows nearly one-third of patients (26.5%) were categorized into the persistent high inflammatory risk group (on-admission and follow-up hs-CRP ≥1.56 mg/L). The prevalence of MACCEs was higher in the persistent high inflammatory risk group (26.8%) than in other groups (*p* < 0.001). Despite statin treatment, a total of 51.7% of post-PCI patients still experienced high cholesterol risk (follow-up LDL-C ≥1.80 mmol/L). The prevalence of persistent high inflammatory risk according to a hs-CRP of 2 mg/L standard is shown in **Supplementary Fig. 4**.

### 3.3 Types of Residual Cholesterol and Cholesterol Risk and Its Association With Recurrent Cardiovascular Events

**Fig. 3.**
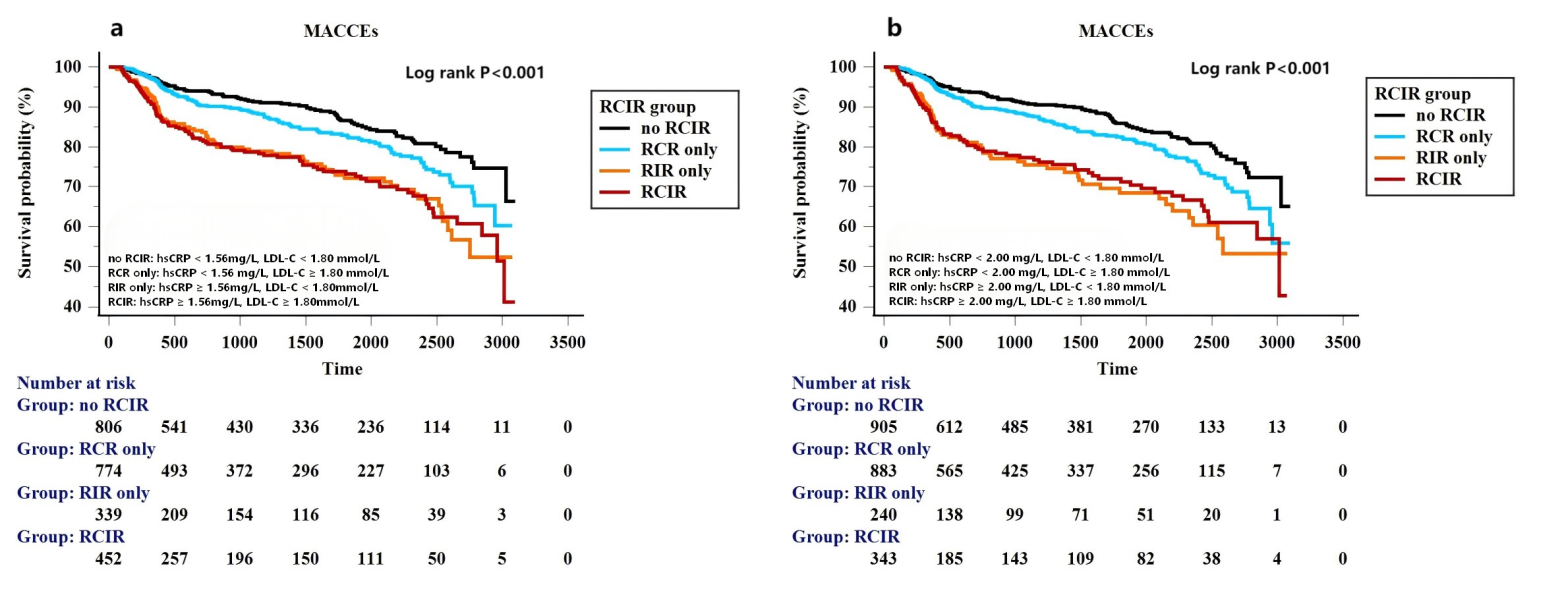
**Kaplan-Meier curves for MACCEs based on different standards (East Asian standard (a), Western standard (b)) for residual cholesterol and inflammatory risk categories**.

**Fig. 4.**
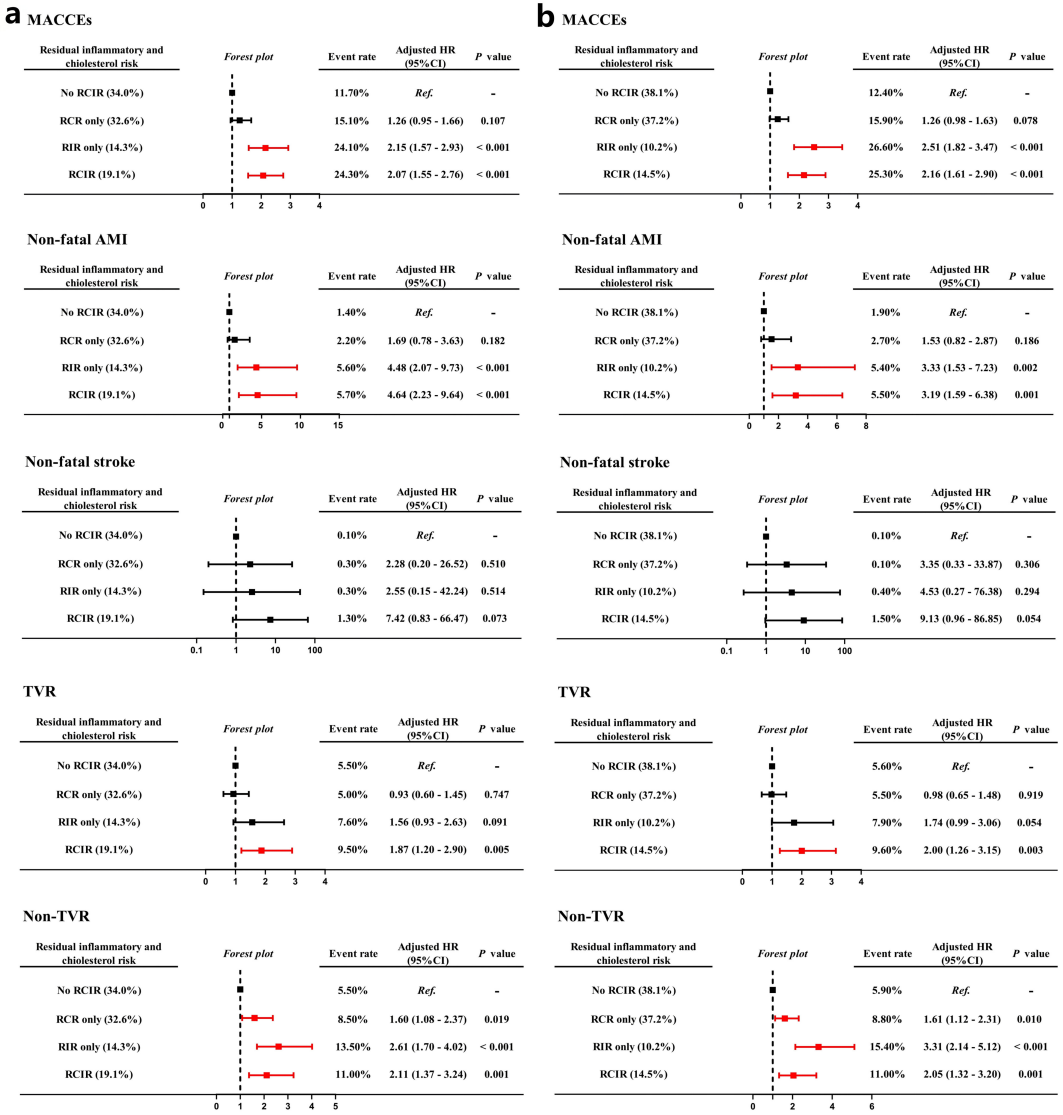
**Forest plots for risks of MACCEs, non-fatal AMI, non-fatal stroke, TVR and non-TVR based on different hs-CRP standards (East Asian standard (a), Western standard (b)) for residual inflammatory and cholesterol risk models**. TVR, target vessel revascularization.

During the follow-up period, there were significant differences in the risk of MACCE across the residual cholesterol or inflammatory risk group, irrespective of whether the hs-CRP threshold value was 1.56 mg/L or 2 mg/L (Fig. 3a,b). Table 3 shows that compared with the no RCIR reference group, the adjusted HR (95% CI) of RCR only, RIR only and the RCIR group for MACCE were 1.26 (0.95–1.66), 2.15 (1.57–2.93) and 2.07 (1.55–2.76) after adjusting for the following confounders: age, male sex, BMI, current smoker, index presentation for PCI, medical history of previous myocardial infarction, coronary revascularization, hypertension and Type 2 diabetes mellitus, the presence of multivessel disease, ACC/AHA defined type B2/C lesions, total stent length, use of ticagrelor and angiotensin blockade at discharge and left ventricular ejection fraction (LVEF) if hs-CRP 1.56 mg/L was used as the high-risk threshold value. Adjusted HR (95% CI) of RCR only, RIR only and RCIR group for MACCE were 1.26 (0.98–1.63), 2.51 (1.82–3.47) and 2.16 (1.61–2.90) if the high-risk hs-CRP cutoff value was 2 mg/L. We further evaluated and compared prognostic implications of residual risks for the incidence of non-fatal AMI, non-fatal stroke, unplanned coronary revascularization according to different high-risk hs-CRP standards. Fig. 4a shows that inflammatory-induced MACCE were more likely to be associated with increased risks of non-fatal AMI (HR 4.48, 95% CI 2.07–9.73, *p* < 0.001), while cholesterol-induced MACCE were more likely to be associated with an increased risk of non-TVR (HR 1.60, 95% CI 1.08–2.37, *p* = 0.019). Associations between residual risk groups according to the Western standard (hs-CRP 2 mg/L) and the risk of MACCE remained consistent after adjusting for confounding factors (Fig. 4b).

**Table 3.** **Determinants of MACCE**.

		Unadjusted model			Adjusted model		
		HR	95% CI	*p* value	HR	95% CI	*p* value
Demographic data							
	Age	0.99	0.98–1.01	0.312	0.99	0.98–1.00	0.136
	Male sex	1.12	0.88–1.43	0.358	0.94	0.68–1.31	0.729
	Body mass index	1.05	1.02–1.08	< **0.001**	1.02	0.99–1.05	0.243
	Current smoker	1.2	0.98–1.47	0.078	1.10	0.84–1.43	0.483
Index presentation				0.969			0.993
	Stable angina	*Ref.*	*Ref.*		*Ref.*	*Ref.*	
	NSTE-ACS	0.99	0.79–1.23	0.916	0.99	0.79–1.25	0.969
	STEMI	0.96	0.72–1.29	0.804	0.98	0.70–1.38	0.904
Medical history							
	Pervious myocardial infarction	1.62	1.26–2.07	< **0.001**	1.34	0.99–1.80	0.056
	Previous coronary revascularization	1.85	1.49–2.29	< **0.001**	1.62	1.26–2.08	< **0.001**
	Hypertension	1.53	1.23–1.90	< **0.001**	1.29	1.00–1.66	**0.046**
	Type 2 diabetes mellitus	1.31	1.08–1.60	**0.007**	1.08	0.88–1.33	0.471
LVEF, %		0.99	0.98–1.01	0.907	1.01	0.99–1.03	0.235
PCI procedural characteristics							
	Multivessel disease	2.14	1.64–2.80	< **0.001**	2.09	1.59–2.75	< **0.001**
	ACC/AHA lesions: type B2 or C	1.09	0.95–1.36	0.346	0.94	0.74–1.19	0.590
	Stent length	1.00	0.99–1.01	0.124	1.00	1.00–1.01	0.065
Medication at discharge							
	Use of ticagrelor	1.29	1.03–1.60	**0.026**	1.18	0.92–1.50	0.194
	Use of angiotensin blockade	1.40	1.14–1.72	**0.001**	1.12	0.89–1.42	0.329
RCIR phenotype (hs-CRP 1.56 mg/L, LDL-C 1.8 mmol/L)				< **0.001**			< **0.001**
	No RCIR	*Ref.*			*Ref.*		
	RCR only	1.36	1.04–1.79	**0.026**	1.26	0.95–1.66	0.107
	RIR only	2.32	1.72–3.12	< **0.001**	2.15	1.57–2.93	< **0.001**
	RCIR	2.36	1.79–3.11	< **0.001**	2.07	1.55–2.76	< **0.001**
RCIR phenotype (hs-CRP 2 mg/L, LDL-C 1.8 mmol/L)							< **0.001**
	No RCIR	*Ref.*			*Ref.*		
	RCR only	1.35	1.06–1.73	**0.017**	1.26	0.98–1.63	0.078
	RIR only	2.62	1.93–3.57	< **0.001**	2.51	1.82–3.47	< **0.001**
	RCIR	2.4	1.81–3.18	< **0.001**	2.16	1.61–2.90	< **0.001**

Adjusted model included age, male sex, body mass index, current smoker, index presentation for PCI, medical history of previous myocardial infarction, coronary revascularization, hypertension, Type 2 diabetes mellitus, left ventricular ejection fraction, multivessel disease, ACC/AHA defined type B2/C lesions, stent length, use of ticagrelor and angiotensin blockade at discharge. *p* value in bold indicate the differences between groups were statistically significant.

### 3.4 The Impact of Persientent Residual Risks and the Associations With Recurrent Cardiovascular Events

**Fig. 5.**
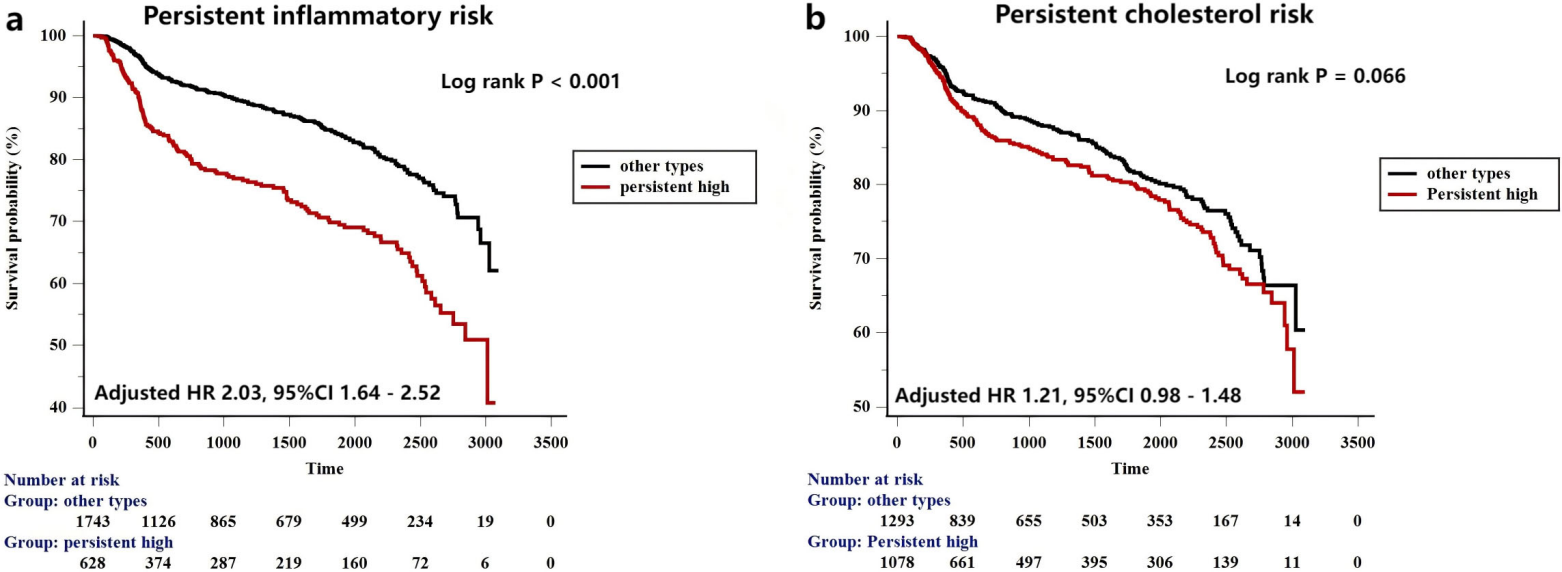
**Kaplan-Meier curves for MACCEs based on the persistent inflammatory (a) and cholesterol risk (b) model**.

To further evaluate the impact of persistent residual risks on prognosis, we also evaluated the prognostic implications of persistent residual risk in the current study (Fig. 5). Persistent high inflammatory risk group was defined as patients with baseline and follow-up hs-CRP ≥1.56 mg/L. Other types of inflammatory groups were defined as the sum of persistent low (baseline and follow-up hs-CRP <1.56 mg/L), attenuated (baseline ≥1.56 mg/L, while follow-up <1.56 mg/L) and fortified (baseline <1.56 mg/L, while follow-up ≥1.56 mg/L) group. Persistent high inflammatory risk was significantly correlated with higher incidence of MACCE (adjusted HR 2.03, 95% CI 1.64–2.52, *p* < 0.001). We failed to find an association between persistent high cholesterol risk and the incidence of MACCE (adjusted HR 1.21, 95% CI 0.98–1.48, *p* = 0.066). Serial measurements of hs-CRP appeared to be more predictive value for MACCE than a single measurement (Persistent high inflammatory risk: adjusted HR 2.03 *vs.* follow-up high inflammatory risk: adjusted HR 1.84 *vs.* baseline high inflammatory risk: adjusted HR 1.40) (Table 4).

**Table 4.** **The predictive value of the baseline, follow-up and persistent high inflammatory risks for MACCE**.

		MACCE events	Unadjusted model			Adjusted model		
		n/N	HR	95% CI	*p* value	HR	95% CI	*p* value
Baseline inflammatory risk								
	Hs-CRP <1.56 mg/L	158/1108	*Ref.*	*Ref.*		*Ref.*	*Ref.*	
	Hs-CRP ≥1.56 mg/L	245/1256	1.41	1.15–1.72	**0.001**	1.40	1.13–1.74	**0.002**
Follow-up inflammatory risk								
	Hs-CRP <1.56 mg/L	211/1580	*Ref.*	*Ref.*		*Ref.*	*Ref.*	
	Hs-CRP ≥1.56 mg/L	192/793	2.00	1.64–2.43	< **0.001**	1.84	1.49–2.27	< **0.001**
Persistent inflammatory risk								
	Other types	234/1743	*Ref.*	*Ref.*		*Ref.*	*Ref.*	
	Persistent high	169/630	2.16	1.77–2.63	< **0.001**	2.03	1.64–2.52	< **0.001**

Adjusted model included age, male sex, body mass index, current smoker, index presentation for PCI, medical history of previous myocardial infarction, coronary revascularization, hypertension, Type 2 diabetes mellitus, left ventricular ejection fraction, multivessel disease, ACC/AHA defined type B2/C lesions, stent length, use of ticagrelor and angiotensin blockade at discharge. *p* value in bold indicate the differences between groups were statistically significant.

Patients were categorized into 4 group according to baseline and follow-up hs-CRP and LDL-C values. Other types included Persistent low, Attenuated and Fortified inflammatory or cholesterol risk group.

## 4. Discussion

The main findings of this study were as follows: (1) Almost half of the PCI-treated patients presented with high cholesterol burden and one-third of PCI-treated patients presented with high inflammatory burden despite lipid-lowering therapies. (2) The inflammatory criteria for high-risk hs-CRP standards may be lower in East Asian patients than their Western counterparts, with a threshold value of 1.56 mg/L in the present study. (3) Cholesterol and inflammation could still be major determinants for recurrent cardiovascular events while hs-CRP seemed to be a stronger predictor than LDL-C in post-PCI patients receiving statins. (4) Serial measurements of hs-CRP levels appear to produce more prognostic values than a single measurement.

### 4.1 The Prevalence of Residual Cholesterol Risk Among East Asians and its Association With Recurrent Cardiovascular Events

Statins remain the cornerstone therapy for the secondary prevention of ASCVD patients due to the pleiotropic effects in lowering cholesterol levels, stabilizing plaques, improving endothelial function and alleviating vascular inflammation [20]. Previous RCTs have shown the effectiveness of statin in reducing future cardiovascular events [2]. However, for advanced ASCVD patients, increased risks for recurrent cardiovascular events can still occur during long-term follow-up despite an early coronary revascularization strategy and guideline-recommended medical therapy, an issue commonly ascribed to the problem of ‘residual risk’ [3, 21, 22]. Cholesterol undoubtedly is a major residual risk factor and was defined as an unachieved LDL-C level goal despite lipid-lowering therapy. While the precise goal for LDL-C remained unknown, current clinical practice guidelines provide Class I recommendations for LDL-C targets of less than 1.80 mmol/L (70 mg/dL) in most patients with atherosclerotic cardiovascular disease [12]. In the present study, the enrolled participants were all post-PCI patients, most of whom received moderate intensity statins (79.5%) at discharge. However, we found that high cholesterol burden could still be present in almost half of the enrolled patients (51.4%) during follow-up, indicating the importance of intensified lipid-lowering therapies in current practice. The concept of ‘the lower, the better’ for LDL-C levels has brought intensified lipid-lowering therapy into clinical practice. Aggressive lipid-lowering therapies have produced positive results and further reduced adverse event rates by 2–15% [5, 23, 24]. Although the predictive value of high cholesterol risk measured by LDL-C for MACCE was mediated by statin treatment in the present study, the beneficial effect of intensified lipid-lowering therapies could not be simply explained by achieving the LDL-C goal. An Asian-specific cohort study focusing on post-PCI patients found that patients receiving high-intensity statins had a lower adjusted risk of major cardiovascular outcomes irrespective of LDL-C target attainment [25]. In addition, we found that RCR was significantly associated with the risk of non-TVR in the present study. The lipid accumulation in non-target vessels that were not severe enough to require intervention during the PCI procedure could be the main cause of recurrent cardiovascular events during the long-term follow-up [26, 27]. Intravascular imaging studies have shown that the benefit of intensified lipid-lowering therapies lies in slowing the plaque progression and lowering the rates of unplanned coronary revascularization [28, 29]. Considering the impact of cholesterol on prognosis and a relatively low percentage of statin-treated patients with achieved LDL-C goals in the current study, intensified lipid-lowering therapy remains necessary in post-PCI patients.

### 4.2 Ethnic Difference in High-Risk hs-CRP Cutoff Value and Its Association With Cardiovascular Risks 

In the past decades, advancement in vascular biology has reshaped our understanding of atherosclerosis. It has shifted from the disease of lipid accumulation in arterial walls to the multifactorial and inflammatory-driven disease. In this novel perspective, inflammation and hyperlipidemia contributed similarly to the initiation and progression of atherosclerosis [30]. The concept of ‘dual targets of inflammatory and cholesterol risk’ has been confirmed in the IMPROVE-IT trial [4]. Increasing evidence from large clinical trials focusing on inflammation among high-risk ASCVD individuals is now emerging. In the Canakinumab Anti-inflammatory Thrombosis Outcomes Study trial (CANTOS trial), participants with a history of myocardial infarction and hs-CRP ≥2 mg/L were randomly allocated to the treatment of canakinumab (an interleukin-1β inhibitor) or placebo group on the basis of standard medical therapy. Compared with placebo, Canakinumab lowered cardiovascular event rates by 15–17%, demonstrating that inhibition of inflammation was a crucial treatment target for atherosclerosis [8]. Recently, reduction of the inflammation with colchicine has emerged as a novel therapeutic option for secondary prevention in ASCVD patients. In the Colchicine Cardiovascular Outcomes Trial (COLCOT trial), patients following a myocardial infarction were randomly assigned to treatment with colchicine 0.5 mg daily or with placebo over a 2-year follow-up, with a 23% relative reduction in the primary endpoint [6]. Similar results were achieved in the Low-Dose Colchicine 2 trial (LoDoCo2 trial), with a 31% risk reduction of the primary endpoint in chronic coronary syndrome patients [7]. However, whether the recognized high-risk hs-CRP threshold (≥2 mg/L) could also be applicable to East Asian patients remains unknown in view of the racial differences in inflammatory activity [9, 10, 11]. The prevalence of high inflammatory risk according to the Western standard in the present study was 24.6%, which was much lower than the data derived from Western registry [31]. Epidemiological studies found that East Asian population exhibit significantly lower median CRP levels (<1 mg/L) compared to Western counterparts (about 3 mg/L) in age or sex–adjusted analysis [11, 32]. The variation in interleukin-6 (IL-6) polymorphism may partly explain the ethnic disparities in inflammatory level, in which the IL-6-174G allele exhibits lower prevalence in Asian population compared to Caucasians, leading to decreased IL-6 expression and consequently reduced hepatic synthesis [33, 34]. In addition, the difference in diet patterns between East Asian and Western population may also partly contribute to ethnic inflammatory disparities. Compared with typical Western diet dominated by processed meats, fried foods and dairy products, traditional East Asian diet shared key anti-inflammatory properties with the Mediterranean diet such as low saturated fat and high Omega-3 fatty acids [35, 36], the latter of which has been experimentally confirmed to inhibit NOD-like receptor family, pyrin domain containing 3 (NLRP3) inflammasome and reduce IL-6 production [37]. Notably, despite lower rates of patients with high inflammatory risk, post-PCI patients in East Asia experienced higher risk of ischemic events caused by persistent high inflammatory risk (baseline and follow-up hs-CRP ≥2 mg/L) than Western counterparts (HR, 2.01 and 1.72, respectively) [38, 39]. Therefore, a tailored hs-CRP cutoff value may be validated in an Asian-specific study. In the present study, the cutoff value for hs-CRP was set at 1.56 mg/L, which was similar to the result of another Asian-based study [40]. In addition to exploring the possibility of lower hs-CRP high-risk standard among East Asian patients, the present study also aimed to compared the separate effect of inflammation and cholesterol on the outcomes of post-PCI patients receiving statin therapy. Whether RCR or RIR dominates in determining prognosis of post-PCI patients constitutes a critical knowledge gap. This creates clinical uncertainty about whether to pursue more intensive lipid-lowering therapy or to initiate anti-inflammatory medications among post-PCI patients already receiving statin therapy. The relative importance of inflammation and cholesterol as determinants of residual cardiovascular risk might have shifted in patients already receiving statin therapy. It was noted that hs-CRP emerged as a stronger predictor for the risk of future cardiovascular events and death than LDL-C among patients receiving statin treatment in a collaborative analysis of three randomized controlled trials [41]. Multivariate Cox regression model analysis showed that RIR was significantly associated with adverse clinical outcomes (mainly triggered by non-fatal AMI in the present study) in this cohort, whereas RCR showed no prognostic value. This finding may indicate inhibiting inflammation may provide greater prognostic benefit than further LDL-C reduction in patients already receiving statin therapy.

### 4.3 Stressing the Importance of Serial Monitoring hs-CRP Value After PCI Treatment

High inflammatory risk continues to persist after PCI treatment, ranging from 18.3% in East Asian populations to 38.0% in Western populations [38, 39]. In the present study, the rate of PCI-treated patients with persistent high inflammatory burden was 18.0% according to the Western standard, which is consistent with previous findings in East Asian populations, showing that almost one-fourth of the PCI-treated populations were under persistent high inflammatory burden. Furthermore, the current study also showed that continuous monitoring of inflammatory indicator could be more valuable than a single measurement in predicting prognosis. The persistent high inflammatory risk was a reliable predictor for prognosis even in patients with baseline LDL-C <1.8 mmol/L, indicating that combination therapy with anti-inflammatory agents should be considered beyond lipid-lowering therapy for patients with high inflammatory risk [42]. The level of inflammation can be dynamically changed over the early phase in unstable patients. A total of 68.4% of enrolled participants presented with ACS, and the inflammatory level could be stabilized after PCI treatment. In addition, high inflammatory risk on admission can also be alleviated by statin treatment at discharge. Hence, serial measurements of hs-CRP should be emphasized following PCI treatment to identify patients with persistent inflammatory risk.

Several limitations should be acknowledged in our study. First, this was a single-center observational retrospective cohort study in which exclusively included post-PCI patients with serial measurements of hs-CRP and LDL-C values, which may unavoidably introduce selection bias in two aspects: (1) Healthcare access disparity: Patients with multiple measurements likely had better care continuity and socioeconomic status, potentially limiting generalizability to disadvantaged populations. (2) Survivorship test: High-risk individuals may die prior to the second measurement, possibly attenuating true risk estimates. Second, the sample size was relatively small, so the exact cutoff value of hs-CRP still needs to be further confirmed in a larger sample size study; Third, the cardiovascular death and stroke rates were relatively low during the follow-up, which may limit the statistical analysis and make it difficult to find an association with the residual risk. Fourth, unmeasured confounders persist despite multivariate Cox regression adjustment; Finally, the intensity and duration of lipid-lowering strategies during the follow-up could not be obtained. Therefore, whether the change in intensity had an impact on the prognostic value of residual risk still needs to be further explored.

## 5. Conclusion

PCI-treated patients receiving statins still presented with a relatively high residual cholesterol and inflammatory burden. The high-risk hs-CRP standard may be lower in East Asian patients than their Western counterparts, with a cutoff value of 1.56 mg/L in present study. Inflammation and cholesterol could be major determinants for recurrent cardiovascular events while hs-CRP seemed to be a stronger predictor than LDL-C in PCI-treated patients after statin treatment.

## 6. Clinical Perspective

Lower hs-CRP cutoff value for East Asian Population: Clinical evidence from Asian or Western registries have substantiated the usefulness of measuring RIR (hs-CRP ≥2 mg/L) in predicting adverse clinical events. Because racial differences in inflammation level exists and East Asian patients usually have lower inflammatory level than Western counterparts, an individualized hs-CRP cutoff value for East Asian population is needed. In our cohort, the high inflammatory risk was set as 1.56 mg/L. The prevalence of patients with high inflammatory risk was 33.4% in our cohort. Serial measurement of hs-CRP levels has shown that a persistent inflammatory risk was a major determinant for adverse clinical events and results in more prognostic value than a single measurement.

Stressing the importance of managing residual inflammatory risk: In the present study, all patients are treated with statin therapy and, thus, the relative importance of inflammation and hyperlipidemia as determinants of residual cardiovascular risk might have shifted. Residual inflammatory risk in the present study seemed to result in more predictive value for future cardiovascular events than the residual cholesterol risk, indicate inhibiting inflammation may provide greater prognostic benefit than further LDL-C reduction in patients already receiving statin therapy.

## Data Availability

The datasets and materials mentioned above are available from the authors upon reasonable request.

## Abbreviations

Hs-CRP, Hypersensitive C-reactive protein; LDL-C, Low-density lipoprotein cholesterol; PCI, Percutaneous coronary intervention; MACCE, Major adverse cardiovascular and cerebrovascular event; AMI, Acute myocardial infarction; TVR, Target vessel revascularization; HR, Hazard ratio; CI, Confidence interval; ASCAD, Advanced atherosclerotic cardiovascular disease; RCR, Residual cholesterol risk; RIR, Residual inflammatory risk; RCIR, Residual cholesterol and inflammatory risk; GF-APT registry, Genetic and platelet function testing for guiding antiplatelet therapy after percutaneous coronary intervention registry; TC, Total cholesterol; TG, Triglyceride; HDL-C, High-density lipoprotein cholesterol; ACS, Acute coronary syndrome; NSTE-ACS, Non-ST-segment elevation acute coronary syndrome; STEMI, ST-segment elevation myocardial infarction; HbA1c, Hemoglobin A1c; BMI, Body mass index; LVEF, Left ventricular ejection fraction; ACEI/ARB, Angiotensin converting enzyme inhibitor/angiotensin receptor blocker; LMCA, Left main coronary artery; LAD, Left anterior descending coronary artery; LCX, Left circumflex coronary artery; RCA, Right coronary artery; CANTOS trial, Canakinumab Anti-inflammatory Thrombosis Outcomes Study trial; COLCOT trial, Colchicine Cardiovascular Outcomes Trial; LoDoCo2 trial, Low-Dose Colchicine 2 trial; IL-6, Interleukin-6; NLRP3, NOD-like receptor family, pyrin domain containing 3.

## Supplementary Material

Supplementary material associated with this article can be found, in the online version, at https://doi.org/10.31083/RCM36438.
