# Supplementary material for: Residual Inflammatory and Cholesterol Risk and the Association With Recurrent Cardiovascular Events in East Asian Patients After Percutaneous Coronary Intervention
Source: Rev Cardiovasc Med. 2025 Sep 19;26(9):36438. doi: 10.31083/RCM36438 (PMC12516752; doi:10.31083/RCM36438)
Supplement: Supplementary file 1 [file 2153-8174-26-9-36438-s1.docx]

**Supplementary files**

**
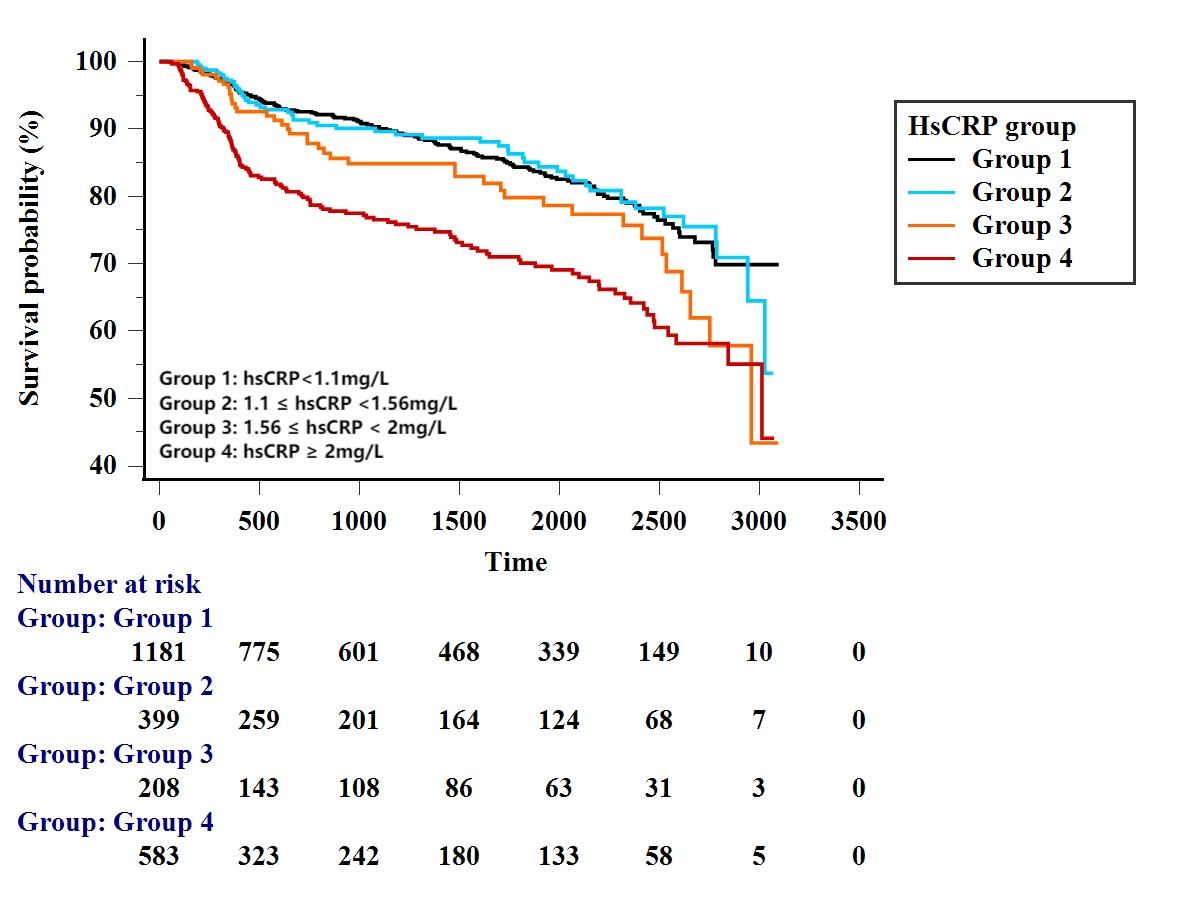
Supplementary Fig. 1.** Kaplan-Meier curves for MACCEs based on high-risk hs-CRP threshold values.

Patients were categorized into 4 groups according to high-risk hs-CRP threshold values derived from tertile stratification (hs-CRP 1.56 mg/L) and RCS (hs-CRP 1.10 mg/L) results. The groups were as follows: Group 1 hs-CRP < 1.1mg/L; Group 2 1.1 ≤ hs-CRP < 1.56 mg/L; Group 3: 1.56 ≤ hs-CRP < 2 mg/L; Group 4: hs-CRP ≥ 2mg/L. Survival analysis showed that compared with Group 1, the MACCEs risk was not significantly increased in Group 2 (HR 0.99, 95%CI 0.73 – 1.35, *P* = 0.962). Differences can be observed in MACCEs risks between Group 3 (HR 1.42, 95%CI 1.01 – 2.01, *P* = 0.045) and Group 4 (HR 2.23, 95%CI 1.79 – 2.80, P < 0.001) if the Group 1 was designated as the reference group for comparison.

**
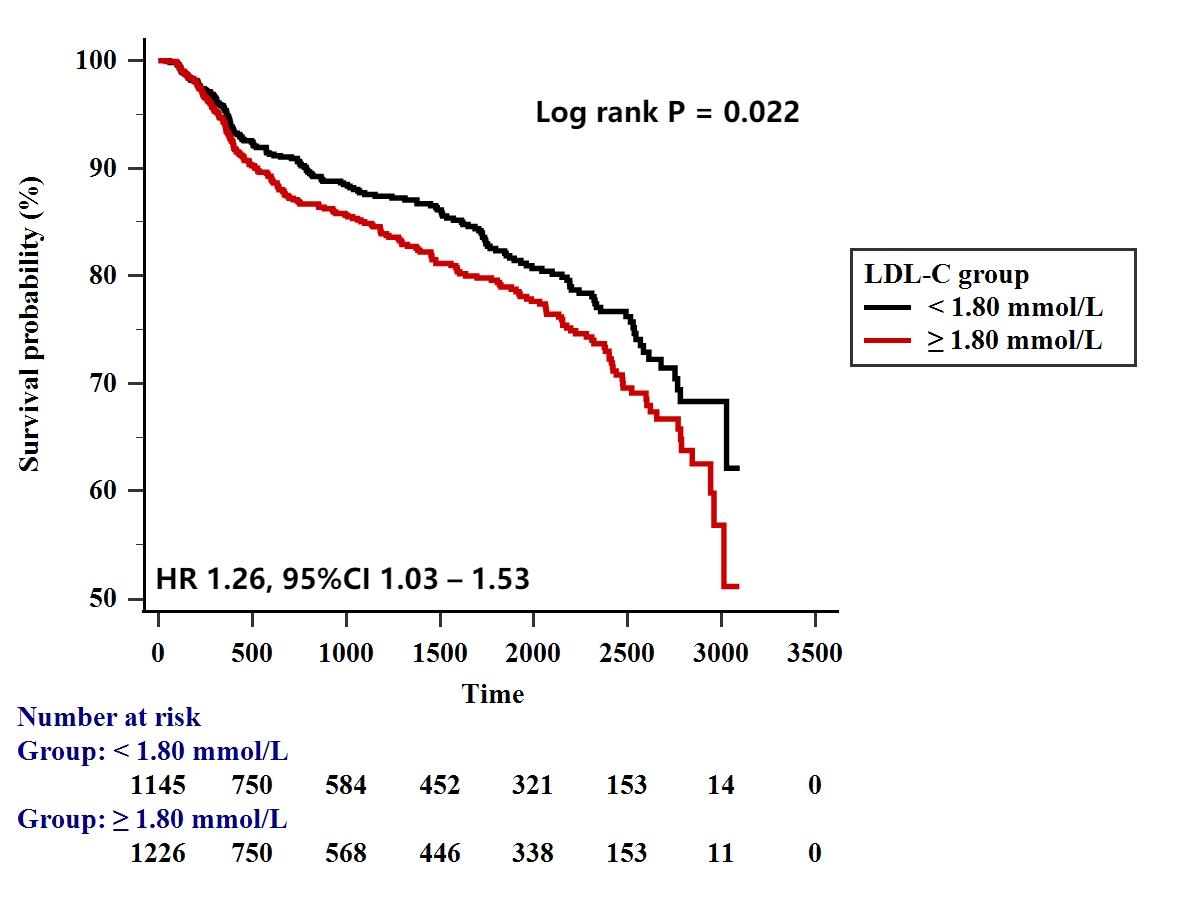
Supplementary Fig. 2.** Kaplan-Meier curves for MACCEs based on the high-risk LDL-C threshold value.

**
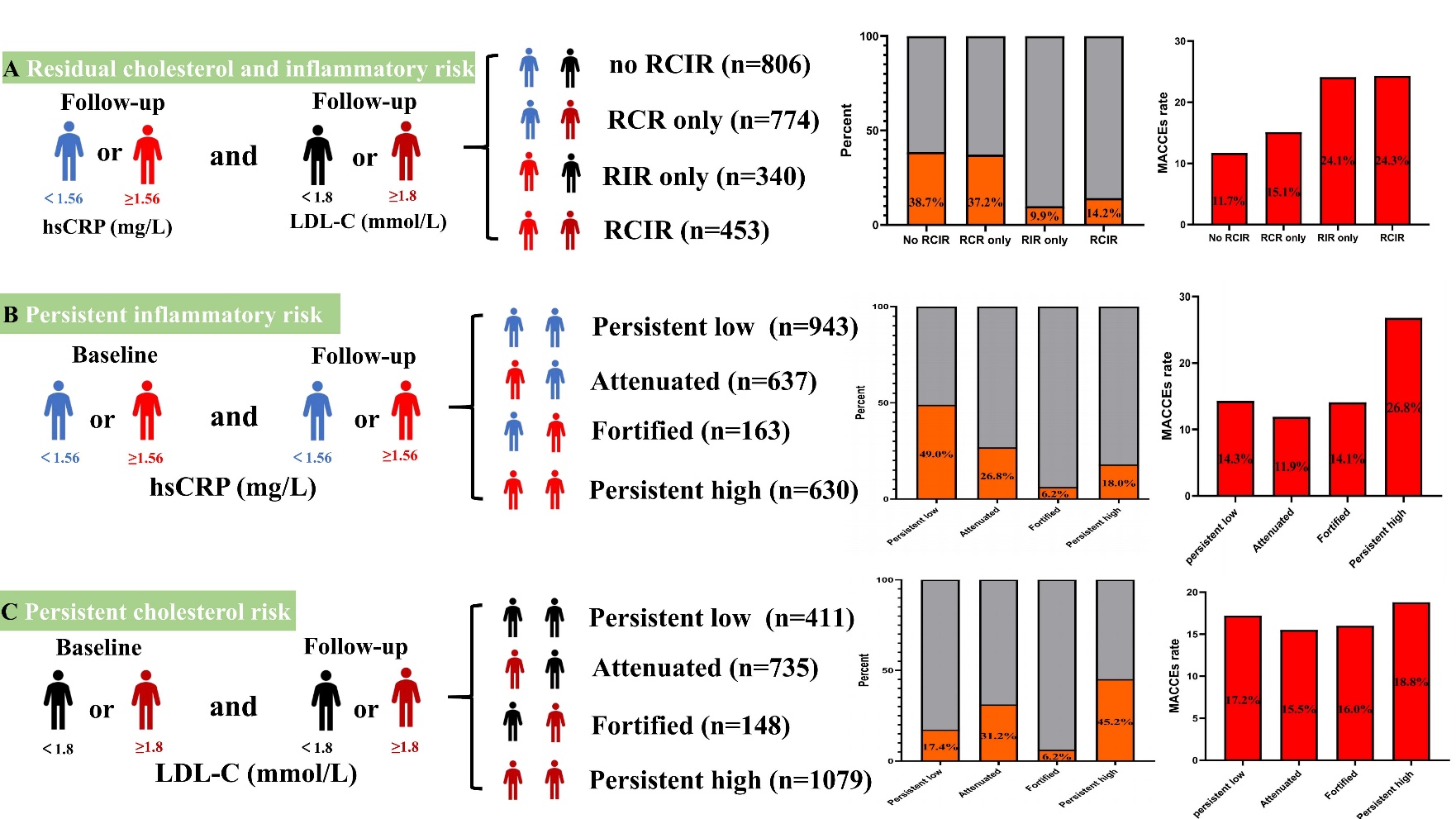
Supplementary Fig. 3.** The distribution of PCI-treated patients and MACCEs rates based on residual inflammatory and cholesterol risk burden (A). The share of persistent inflammatory risk and MACCEs rates among PCI-treated patients stratified by hs-CRP ≥ 1.56 mg/L criterion (B). The share of persistent cholesterol risk and MACCEs rates among PCI-treated patients stratified by LDL-C ≥ 1.8 mmol/L criterion (C).

**
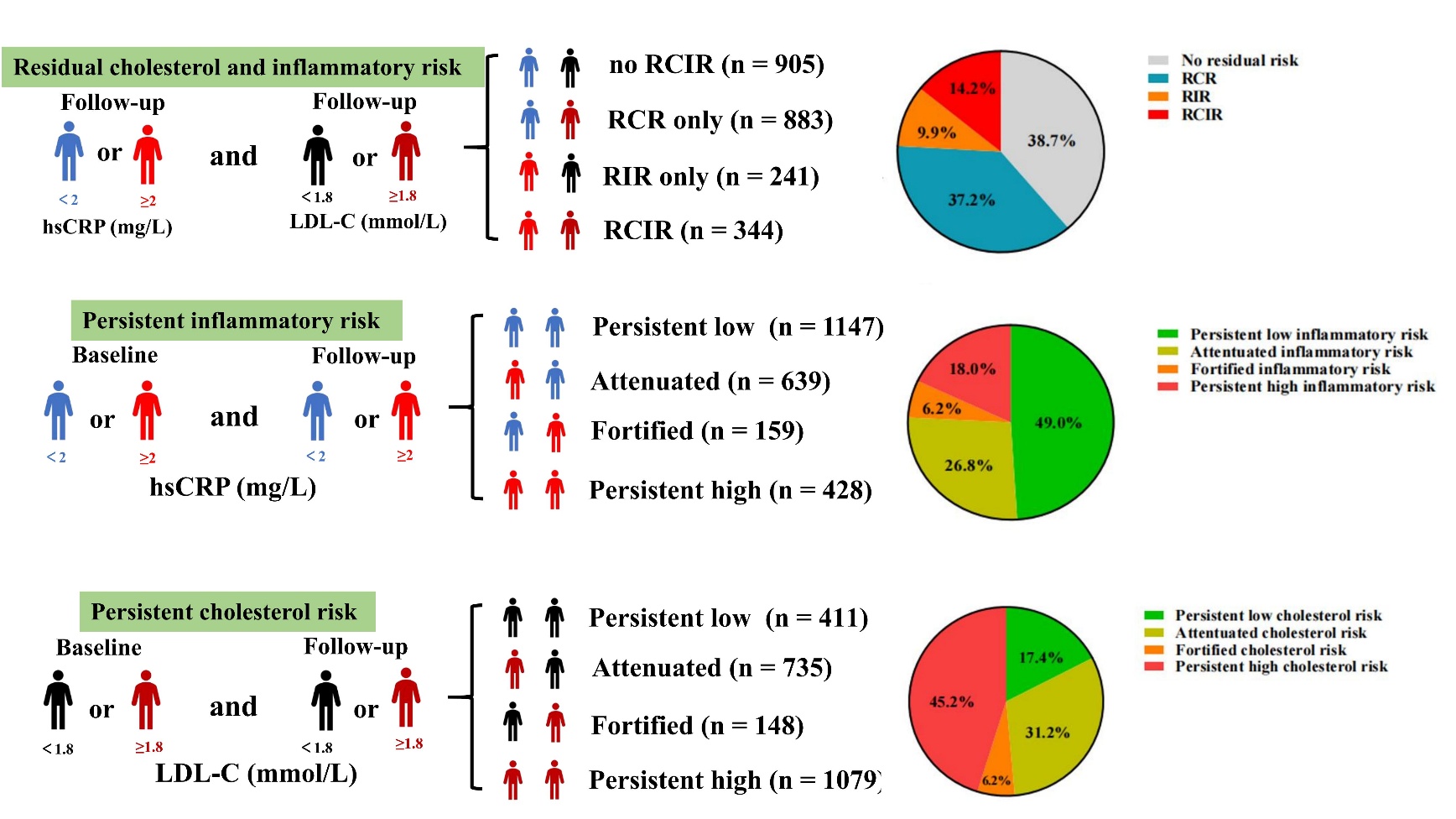
Supplementary Fig. 4.** The distribution of PCI-treated patients based on residual inflammatory and cholesterol risk burden (A). The share of persistent inflammatory risk and MACCEs rates among PCI-treated patients stratified by hs-CRP ≥ 2 mg/L criterion (B). The share of persistent cholesterol risk and MACCEs rates among PCI-treated patients stratified by LDL-C ≥ 1.8 mmol/L criterion (C).
